# TET2 Expression in Bone Marrow Mononuclear Cells of Patients with Myelodysplastic Syndromes and Its Clinical Significances

**DOI:** 10.3969/j.issn.2095-3941.2012.01.006

**Published:** 2012-03

**Authors:** Wei Zhang, Zong-hong Shao, Rong Fu, Hua-quan Wang, Li-juan Li, Jun Wang, Wen Qu, Yong Liang, Guo-jin Wang, Xiao-ming Wang, Yuhong Wu, Hong Liu, Jia Song, Jing Guan, Li-min Xing

**Affiliations:** Department of Hematology, Tianjin Medical University General Hospital, Tianjin 300052, China

**Keywords:** myelodysplastic syndrome, TET2 gene, clinical features

## Abstract

**Objective:**

To investigate the expression of TET2 mRNA and protein in the bone marrow mononuclear cells (BMMNC) of patients with myelodysplastic syndrome (MDS) and its clinical significance.

**Methods:**

The expression of TET2 mRNA and protein in bone marrow mononuclear cells (BMMNC) of 32 patients with MDS and 20 healthy donors was examined by qPCR and Western blot.

**Results:**

The expression of TET2 mRNA in BMMNC was down-regulated in MDS patients compared with the donor group [(0.41±0.28)% *vs.* (1.07±0.56)%] (*P*<0.001). Compared with lower expression group (TET2<0.4) [(6.53±6.17)%], patients with higher expression of TET2 (≥0.4) presented significantly lower proportion of bone marrow blasts [(1.21±1.56)%] (*P*<0.05). The expression of TET2 mRNA in BMMNC of MDS patients was inversely correlated with malignant clone burden (*r*=-0.398, *P*<0.05) and IPSS (*r*=-0.412, *P*<0.05). The expression of TET2 protein was down-regulated in MDS patients compared with that in the donor group.

**Conclusions:**

The mRNA and protein expression of TET2 in BMMNC of MDS patients is decreased, which might be useful as an important parameter for the evaluation of MDS clone burden.

## Introduction

Myelodysplastic syndrome (MDS) is a heterogeneous group of malignant disorders characterized by ineffective hematopoiesis in a single or several hematopoietic cell lineages, that lead to abnormal proliferation and differentiation of these lineages, with a high risk of evolving into acute myeloid leukemia (AML) ^[^^1^^, ^^2^^]^. It has been found that MDS patients present abnormal expression of some oncogenes and tumor suppressor genes. TET2, a member of the ten-eleven-translocation (TET) family genes, located in chromosome 4q24, can mutate in various hematopoietic disorders, including myeloprolieferative neoplasms (MPNs), MDS, acute myeloid leukemia, and chronic myelomonocytic leukemia ^[^^3^^-^^12^^]^. This study investigated the mRNA and protein expression of TET2 in the bone marrow mononuclear cells (BMMNC) of patients with MDS and analyzed the relationship between TET2 mRNA expression and patients’ clinical features.

## Materials and Methods

### Patients

Written informed consent was obtained from all patients before they entered the trail. Thirty-two patients with MDS diagnosed according to WHO classification ^[^^13^^]^ in our department from May 2010 to December 2011 were enrolled in this study. There were 5 refractory anemia (RA) cases, 1 refractory anemia with ring sideroblasts (RARS), 12 cases of refractory cytopaenia with multilineage dysplasia (RCMD), 4 refractory anemia cases with excess blasts I (RAEB-I) and 10 refractory anemia cases with excess blasts, II (RAEB-II) including 20 males and 12 females, with a median age of 60 years. Based on IPSS, these patients were divided into 2 groups, 11 in low-risk and 21 in high-risk group. Twenty healthy donors were also studied.

### IPSS scoring

Prognostic scores were calculated based on marrow blast percentage, karyotype, and the number of cytopenias according to the IPSS. Marrow blast percentages were obtained by light microscopy as described in “Morphologic assessment of marrow.” Cytopenias were defined as hemoglobin value less than 10 g/dL, absolute neutrophil count (ANC) less than 1.5×10^9^/L, and platelet values less than 100×10^9^/L. Karyotypes were divided into subgroups of good [normal, -Y, del(5q), del(20q)], poor (3 or more abnormalities or chromosome 7 abnormalities), and intermediate (all remaining karyotypes) risk.

### qPCR analysis

One milliliter bone marrow sample was obtained and anti-coagulated with 2% EDTA, 30 mL hemolysin (1:10 diluted)was added, and then stored in dark place for 10 min. The sample was then cetrifugated in 1000 *r*/min for 10 min, the sedimentation was washed with PBS. The sedimentation was the bone marrow mononuclear cell (BMMNC). Total RNA was extracted using Trizol (Invitrogen, Carlsbad, CA), and cDNA was generated using SuperScript III RT kit (Invitrogen, Carlsbad, CA). PCR cycling conditions were initial denaturation at 50°C for 2 min, then 95°C for 5 min followed by 40 cycles at 95°C for 15 sec, at 60°C for 60 sec. SYBR Green qPCR Master MIX (2×) (Affymetrix, USB, USA) was used in PCR. Primers used are listed as follows: TET2: forward 5’-GCC AAG TCG TTA TTT GAC CA -3’, reverse 5’-CTG AAG AAG TTG TTT GCT GCT CTA -3’; β-actin: forward 5’-CTA CAA TGA GCT GCG TGT GGC-3’, reverse 5’-CAG GTC CAG ACG CAG GAT GGC-3’ (synthesized by Shanghai Biotech Co., Ltd, China). Applied Bio-Rad CFX Manager software, each group relative quantitative using 2^-ΔΔCt^ values​?: ??C​: ΔΔC_t_= (C_ttarget_−C_tβ-actin_)_target_−(C_ttarget_−C_tβ-actin_)_ctrl_.

### Western blot

Total protein was extracted using RIPA (Biotech, Beijing). Total protein concentration was determined using the commercial Bradford reagent assay (Bio-Rad, Hercules, CA). Fifty µg of total protein was used for the detection of TET2/GAPDH for each of the treatments. Samples were first boiled in sample buffer (125 mM Tris-HCl pH 6.8, 1% v/w SDS, 10% v/v glycerol, 0.1% bromophenol blue, 2% v/v 2beta-mercaptoethanol) for 5 min and separated by 10% SDS-PAGE. Then, the gels were transferred to PVDF membranes (PE, USA) using a Trans-Blot Cell system (Bio-Rad, Hercules, CA) in transfer buffer (25 mM Tris, 190 mM glycine, 10% methanol) at 80 V for 1 h. After transfer, incubate membrane in blocking buffer for 1 h at room temperature. Wash 3 times for 5 min each with 15 mL of PBS/T. Then, incubate membrane and rabbit anti-human TET2 antibody (Sta. Cruz, CA) (1:100) in 10 mL primary antibody dilution buffer with gentle agitation overnight at 4°C. After washing, incubate membrane with appropriate HRP-conjugated secondary antibody (1:5000) to detect biotinylated protein markers in 10 mL of blocking buffer with gentle agitation for 1 h at room temperature. The signals were detected by enhanced chemiluminescence using the supersignal system (Pierce Rockford, IL) and quantified by densitometry. As a control, GAPDH was simultaneously detected, using a mouse anti-human GAPDH antibody. The antibody was diluted 1:1000 and developed using the secondary antibody and chemiluminescence system previously described.

### Karyotype analysis

Five mL fresh bone marrow as studied sample was obtained from the patients and controls, and put in 1640 medium with heparin. Then (1-2)×10^6^/mL cells were took out and cultured in 1640 medium supplemented with 20% fetal bovine serum. The cultures were maintained at 37°C in a moist atmosphere containing 5% CO_2_ for 24 h. Then colchicines (final concentration 0.05 µg/mL) was added to the sample. After being reacted at 37°C for 1 h, the cell samples were mixed with 8 mL of 0.075 M 37°C KCL for 30 min. The cells were fixed with stationary liquid for 3 times, then conserved in 4°C refrigerator and were treated for R-binding. Twenty metaphases were evaluated.

### Statistical analysis

The software of SPSS 18.0 was used. Measurement data were displayed in the form of mean ± SD. T-test was used for comparing means. If the distribution wasn’t normal, measurement data were displayed in the form of median value and range and nonparametric test (Mann-Whitney U) was used for comparing median value. One-way analysis of variance was used to analyze significance between groups. Spearman bivariate correlation analysis was used for analyzing correlation. *P*<0.05 indicated statistical significance.

## Results

### Expression of TET2 mRNA in the BMMNC of patients with MDS and normal controls

The expression of TET2 mRNA in the BMMNC of 32 MDS patients and 20 healthy donors as control group were measured. Compared with that in the control group, the expression of TET2 mRNA was lower in MDS patients [(0.41±0.28) *vs.* (1.07±0.56)] (*P*<0.001).

### Expression of TET2 mRNA in the BMMNC of patients with different MDS types

**Figure 1:**
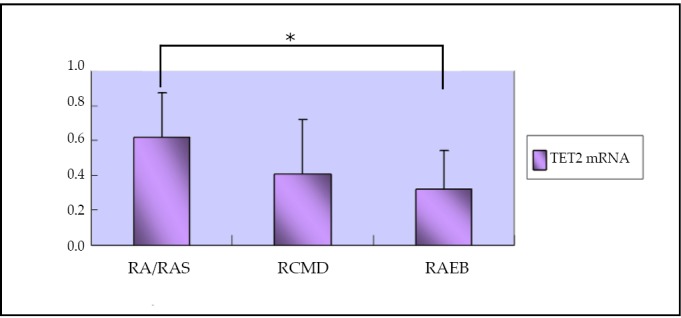
Expression of TET2 mRNA in the BMMNC of patients with different MDS types. * comparison between RA/RARS and RAEB patients, *P*<0.05.

The average expression of TET2 mRNA was 0.63 (0.28-1.01) in RA and RAS patients, 0.39 (0.09-1.23) in RCMD patients, and 0.27 (0.11-0.91) in RAEB patients. There was significant difference between RA/RAS and RAEB groups (*P*<0.05, **Figure 1**).

### Expression of TET2 protein in the BMMNC of patients with MDS and controls

**Figure 2:**
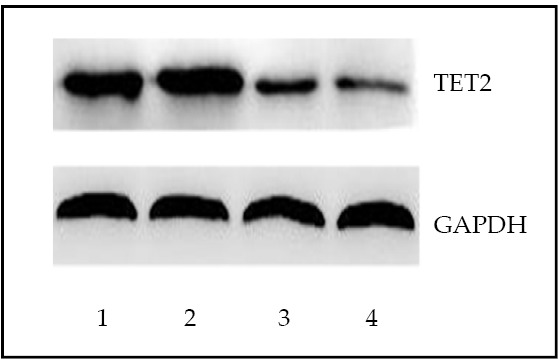
Detection of TET2 protein by Western blot in bone marrow mononuclear cells from MDS patients and healthy controls. Lanes1, 2: normal control; Lanes 3, 4: MDS patients.

The expression of TET2 protein in MDS patients was lower than that of normal controls (*P*<0.01, **Table 1**, **Figure 2**).

**Table 1:** The expression of TET2 protein in MDS patients and normal controls (M, range).

Group	No. of patients	Expression of TET2
MDS	10	0.46, 0.34-0.81*
Healthy donors	5	1.02, 0.89-1.12

* compared with normal controls, *P*<0.01.

### Relationship between the expression of TET2 mRNA and the proportion of the bone marrow blasts

Analysis of the clinical data of MDS patients found that patients with high expression of TET2 in BMMNC usually presented lower percentage of bone marrow blast. High expression of TET2 (≥0.4) was found in 13 patients whose average proportion of bone marrow blasts was (1.21±1.56)%, and lower expression of TET2 (<0.4) was found in 12 patients whose average proportion of blasts was (6.53±6.17)% (*P*<0.05).

### Relationship between the expression of TET2 mRNA and chromosome karyotype and burden of malignant clone

There were 11 patients with normal chromosome karyotype, with the average expression of TET2 0.38 (0.15-1.01); 21 patients with abnormal chromosome karyotype, and the average expression of TET2 was 0.33 (0.09-1.23). There was no statistical significance (*P*>0.05). The IPSS criteria were used to distinguish good, intermediate, and poor risk cytogenetics. There was significant difference between good and poor groups (*P*<0.05, **Table 2**).

**Table 2:** The expression of TET2 gene in patients with different karyotype (M, range).

Karyotype	No. of patients	Expression of TET2
Good	12	0.41, 0.15-1.23
Intermediate	8	0.36, 0.25-0.91
Poor	12	0.23, 0.09-0.68*

* compared with good group, *P*<0.05

To analyze the correlation between the expression of TET2 and burden of malignant clone, the proportion of abnormal karyotype cells was regarded as burden of malignant clone. Expression of TET2 gene decreased when the burden of malignant clone increased. So they were inversely correlated (*r*=-0.398, *P*<0.05).

### Relationship between the expression of TET2 mRNA and IPSS

According to patients’ clinical data, IPSS was counted. Expression of TET2 gene and IPSS was also inversely correlated (*r*=-0.412, *P*<0.05).

## Discussion

Recent studies showed that TET2 was the most frequently mutating gene in MDS known so far ^[^^3^^, ^^5^^]^. Molecular and cytogenetic approaches can identify the ten-eleven translocation 2 (TET2) gene in a common 500-kb minimal deleted region. The TET2 gene contains 11 exons spreading over 150 kb ^[^^3^^]^. The 2002 amino acids of TET2 protein exhibits two evolutionary conserved regions: one region located from amino acid 1134 to amino acid 1444 and the other region located near the carboxyterminal end from amino acid 1842 to amino acid 1921 that is related to the hydroxylase family and depends on iron and 2-oxoglutarate ^[^^14^^, ^^15^^]^. TET2 mutations were also reported in other myeloid malignancies ^[^^3^^, ^^6^^, ^^16^^]^. Langemeijer et al.^[^^17^^]^ conducted SNP array–based genomic profiling and genomic sequencing in 102 individuals with MDS and identified acquired deletions and missense and nonsense mutations in the TET2 gene in 26% of these individuals. With allele-specific assays, TET2 mutations were detected in most of the bone marrow cells. In addition, the TET2 mutations occurred in various cell differentiation stages including CD34^+^ progenitor cells, suggesting that they mutated early during disease evolution. In healthy tissues, TET2 expression was shown to be elevated in hematopoietic cells with highest expression in granulocytes, in line with a function in myelopoiesis.

Jankowska et al. ^[^^7^^]^ found that the highest expression of TET2 mRNA was in CD33^+^ myeloid cells or CD14^+^ monocytes; as well as the CD34^+^ cells of healthy persons. Decreased TET2 expression was reported in patients with hematologic malignancy ^[^^7^^]^, which was consistent with our result.

Kosmider et al. ^[^^18^^]^ showed that frameshift, nonsense or missense mutations, or defects in gene structure were identified in 22 (22.9%) of 96 MDS patients. The 5-year OS was 76.9% in patients with mutation *vs.* 18.3% in those without mutation (*P*<0.005). The 3-year leukemia free survival was 89.3% in mutated *vs.* 63.7% in unmutated patients (*P*<0.035). By univariate analysis, the absence of TET2 mutation was associated with a 4.1-fold increased risk of death (*P*<0.009). By multivariate analysis adjusted for age, International Prognostic Scoring System score, and transfusion requirement, the presence of TET2 mutation remained an independent factor of favorable prognosis. An additional study by Smith et al. ^[^^19^^]^ found no prognostic significance for TET2 mutations, even when clustered according to World Health Organization subtypes, International Prognostic Scoring System score, cytogenetic status, or transformation to AML.

Recent studies showed that TET genes were identified to catalyze the conversion of cytosine-5 methylation to 5-hydroxymethyl-cytosine, which is an intermediate form potentially involved in demethylation. And there was a correlation between low genomic 5-hydroxymethyl-cytosine and TET2 mutation in patients with myeloid malignancies^[^^20^^-^^25^^]^.

This study found that TET2 mRNA and TET2 protein expression was lower in the BMMNC of MDS patients than in normal controls, and TET2 mRNA relative expression was inversely correlated with IPSS and the burden of malignant clone, suggesting that TET2 was a protective gene for MDS patients and an indicator for evaluating the state of illness. More investigation about the function of TET2 in MDS and the mechanisms in the myeloid malignancies were warranted.

## References

[1] Shi J Shao ZH Liu H Transformation of myelodysplastic syndromes into acute myeloid leukemias. Chin Med J 2004; 117: 963-967 15265365

[2] Nimer SD Myelodysplastic syndromes. Blood 2008; 111: 4841-4851 18467609 10.1182/blood-2007-08-078139

[3] Delhommeau F Dupont S Della Valle V Mutation in TET2 in myeloid cancers. N Engl J Med 2009; 360: 2289-2301 19474426 10.1056/NEJMoa0810069

[4] Couronné L Lippert E Andrieux J Analyses of TET2 mutations in post-myeloproliferative neoplasm acute myeloid leukemias. Leukemia 2010; 24: 201-203 19710701 10.1038/leu.2009.169

[5] Mullighan CG TET2 mutations in myelodysplasia and myeloid malignancies. Nat Genet 2009; 41: 766-767 19557078 10.1038/ng0709-766

[6] Abdel-Wahab O Mullally A Hedvat C Genetic characterization of TET1, TET2, and TET3 alterations in myeloid malignancies. Blood 2009; 114: 144-147 19420352 10.1182/blood-2009-03-210039 PMC2710942

[7] Jankowska AM Szpurka H Tiu RV Loss of heterozygosity 4q24 and TET2 mutations associated with myelodysplastic/myeloproliferative neoplasms. Blood 2009; 113: 6403-6410 19372255 10.1182/blood-2009-02-205690 PMC2710933

[8] Mohamedali AM Smith AE Gaken J Novel TET2 mutations associated with UPD4q24 in myelodysplastic syndrome. J Clin Oncol 2009; 27: 4002-4006 19528370 10.1200/JCO.2009.22.6985

[9] Tefferi A Levine RL Lim KH Frequent TET2 mutations in systemic mastocytosis: clinical, KITD816V and FIP1L1-PDGFRA correlates. Leukemia 2009; 23: 900-904 19262599 10.1038/leu.2009.37 PMC4654631

[10] Hussein K Abdel-Wahab O Lasho TL Cytogenetic correlates of TET2 mutations in 199 patients with myeloproliferative neoplasms. Am J Hematol 2010; 85: 81–83 19957346 10.1002/ajh.21562 PMC4654625

[11] Hussein K Van Dyke DL Tefferi A Conventional cytogenetics in myelofibrosis: literature review and discussion. Eur J Haematol 2009; 82: 329-338 19141119 10.1111/j.1600-0609.2009.01224.x

[12] Tefferi A Lim KH Abdel-Wahab O Detection of mutant TET2 in myeloid malignancies other than myeloproliferative neoplasms: CMML, MDS, MDS/MPN and AML. Leukemia 2009; 23: 1343-1345 19295549 10.1038/leu.2009.59 PMC4654626

[13] Vardiman JW Harris NL Brunning RD The World Health Organization (WHO) classification of the myeloid neoplasms. Blood 2002; 100: 2292-2302 12239137 10.1182/blood-2002-04-1199

[14] Tahiliani M Koh KP Shen Y Conversion of 5-methylcytosine to 5-hydroxymethylcytosine in mammalian DNA by MLL partner TET1. Science 2009; 324: 930-935 19372391 10.1126/science.1170116 PMC2715015

[15] Nibourel O Kosmider O Cheok M Incidence and prognostic value of TET2 alterations in de novo acute myeloid leukemia achieving complete remission. Blood 2010; 116: 1132-1135 20489055 10.1182/blood-2009-07-234484

[16] Tefferi A Pardanani A Lim KH TET2 mutations and their clinical correlates in polycythemia vera, essential thrombocythemia and myelofibrosis. Leukemia 2009; 23: 905-911 19262601 10.1038/leu.2009.47 PMC4654629

[17] Langemeijer SM Kuiper RP Berends M Acquired mutations in TET2 are common in myelodysplastic syndromes. Nat Genet 2009; 41: 838-842 19483684 10.1038/ng.391

[18] Kosmider O Gelsi-Boyer V Cheok M TET2 mutation is an independent favorable prognostic factor in myelodysplastic syndromes (MDSs). Blood 2009; 114: 3285-3291 19666869 10.1182/blood-2009-04-215814

[19] Smith AE Mohamedili AM Kulasekararaj A Next-generation sequencing of TET2 gene in 355 MDS and CMML patients reveals low-abundance mutant clones with early origins, but indicates no definite prognostic value. Blood 2010; 116: 3923-3932 20693430 10.1182/blood-2010-03-274704

[20] Mohr F Döhner K Buske C TET genes: new players in DNA demethylation and important determinants for stemness. Exp Hematol 2011; 39: 272-281 21168469 10.1016/j.exphem.2010.12.004

[21] Tahiliani M Koh KP Shen Y Conversion of 5-methylcytosine to 5-hydroxymethylcytosine in mammalian DNA by MLL partner TET1. Science 2009; 324: 930-935 19372391 10.1126/science.1170116 PMC2715015

[22] Pollyea DA Raval A Kusler B Impact of TET2 mutations on mRNA expression and clinical outcomes in MDS patients treated with DNA methyltransferase inhibitors. Hematol Oncol 2011; 29: 157-160 21922510 10.1002/hon.976

[23] Koh KP Yabuuchi A Rao S Tet1 and Tet2 Regulate 5-Hydroxymethylcytosine Production and Cell Lineage Specification in Mouse Embryonic Stem Cells. Cell Stem Cell 2011; 8: 200-213 21295276 10.1016/j.stem.2011.01.008 PMC3134318

[24] Itzykson R Kosmider O Cluzeau T Impact of TET2 mutations on response rate to azacitidine in myelodysplastic syndromes and low blast count acute myeloid leukemias. Leukemia 2011; 25: 1147-1152 21494260 10.1038/leu.2011.71

[25] Konstandin N Bultmann S Szwagierczak A Genomic 5-hydroxymethylcytosine levels correlate with TET2 mutations and a distinct global gene expression pattern in secondary acute myeloid leukemia. Leukemia 2011; 25: 1649-1652 21625234 10.1038/leu.2011.134

